# Our Table of Societies

**Published:** 1868-11

**Authors:** 


### OUR TABLE OF SOCIETIES.

As a matter of convenience to the profession we have for several years published in the pages of the REGISTER a list of all the Dental societies in the United States. We have endeavored in its corrections to follow the changes occurring in the officers of all these different societies. The number has so much increased that this is not an easy task, and without the coöperation of the different societies almost impossible. We therefore earnestly request the officers of all the societies to give us correct information in regard to their respective organizations, of the time and place of meeting, and especially the names of officers and their address. By a little attention on the part of those interested, our list can be kept far more accurate, than it is possible to do otherwise.

